# Human bladder cancer invasion model using rat bladder in vitro and its use to test mechanisms and therapeutic inhibitors of invasion

**DOI:** 10.1054/bjoc.2000.1641

**Published:** 2001-02

**Authors:** C Fujiyama, A Jones, S Fuggle, R Bicknell, D Cranston, A L Harris

**Affiliations:** Molecular Oncology Unit, ICRF, Institute of Molecular Medicine, John Radcliffe Hospital, Oxford, OX3 9DS; Department of Urology, Churchill Hospital, Oxford, OX3 7LJ, UK; Nuffield Department of Surgery, University of Oxford, John Radcliffe Hospita, Oxford, OX3 9DU, UK

**Keywords:** invasion, in vitro assay, bladder cancer, suramin, N-acetylcysteine

## Abstract

As well as being a passive support, the extracellular matrix also regulates key biological processes such as invasion, differentiation and angiogenesis. We have therefore developed an in vitro model of bladder cancer invasion using de-epithelialized rat bladder to allow for tumour cell–extracellular matrix interactions. Onto this we have seeded a panel of human bladder cancer cell lines (RT4, RT112, 253J and EJ28 (T24)) representing progression from well to poorly differentiated phenotypes and used as models of superficial to invasive bladder cancer. The better differentiated cell lines RT4 and RT112 reproducibly grew as stratified epithelium, whereas poorly differentiated EJ28 cells invaded across a broad front. Invasion was not simply related to proliferation rate, measured either as doubling time on plastic (non-invasive 253J and invasive EJ28 having the same doubling time) or by Ki-67 proliferation index within the model. We used the model to test the ability of 4 compounds that interfere with tumour cell–extracellular matrix interactions (suramin, N-acetylcysteine and the urokinase plasminogen activator pathway antagonists Å5 compound and monoclonal antibody Mab 3936) to inhibit invasion. At non-toxic concentrations, all significantly inhibited invasion (*P*< 0.05), although to varying degrees, suramin and Å5 almost completely and N-acetylcysteine the least. In conclusion, this model shows the urokinase system is important for bladder invasion and can be used to investigate other mechanisms of bladder cancer invasion and also for the testing of intravesical drugs. © 2001 Cancer Research Campaign http://www.bjcancer.com


*British Journal of Cancer* (2001) **84**(4), 558–564
© 2001 Cancer Research Campaign
doi: 10.1054/ bjoc.2000.1641, available online at http://www.idealibrary.com on IDEAL®

### Human bladder cancer invasion model using rat bladder in vitro and its use to test mechanisms and therapeutic inhibitors of invasion

**C Fujiyama*[,1,3], A Jones*[,1,2,3], S Fuggle[3], R Bicknell[1], D Cranston[2] and AL Harris[1]**

[1]Molecular Oncology Unit, ICRF, Institute of Molecular Medicine, John Radcliffe Hospital, Oxford OX3 9DS; [2]Department of Urology, Churchill Hospital, Oxford OX3 7LJ; Nuffield Department of Surgery, University of Oxford, John Radcliffe Hospita, Oxford OX3 9DU, UK

**Summary** As well as being a passive support, the extracellular matrix also regulates key biological processes such as invasion, differentiation and angiogenesis. We have therefore developed an in vitro model of bladder cancer invasion using de-epithelialized rat bladder to allow for tumour cell–extracellular matrix interactions. Onto this we have seeded a panel of human bladder cancer cell lines (RT4, RT112, 253J and EJ28 (T24)) representing progression from well to poorly differentiated phenotypes and used as models of superficial to invasive bladder cancer. The better differentiated cell lines RT4 and RT112 reproducibly grew as stratified epithelium, whereas poorly differentiated EJ28 cells invaded across a broad front. Invasion was not simply related to proliferation rate, measured either as doubling time on plastic (non-invasive 253J and invasive EJ28 having the same doubling time) or by Ki-67 proliferation index within the model. We used the model to test the ability of 4 compounds that interfere with tumour cell–extracellular matrix interactions (suramin, N-acetylcysteine and the urokinase plasminogen activator pathway antagonists Å5 compound and monoclonal antibody Mab 3936) to inhibit invasion. At non-toxic concentrations, all significantly inhibited invasion ($P < 0.05$), although to varying degrees, suramin and Å5 almost completely and N-acetylcysteine the least. In conclusion, this model shows the urokinase system is important for bladder invasion and can be used to investigate other mechanisms of bladder cancer invasion and also for the testing of intravesical drugs. © 2001 Cancer Research Campaign   htt://www.bjcancer.com

**Keywords:** invasion; in vitro assay; bladder cancer; suramin; N-acetylcysteine

Bladder cancer accounts for approximately 8% and 6% of all new male cancers and 4% and 3% of male cancer deaths each year in the United Kingdom and the USA respectively (Silverman et al, 1999). Three quarters of all cases present as superficial disease and whilst half of these cases are cured by simple treatment, about half will develop recurrences (Gilbert et al, 1978). The field change theory of recurrence proposes that each recurrence has arisen de novo from a generalized precancerous urothelium (Raghavan et al, 1990; Harris and Neal, 1992). The clonal origin of recurrence (Sidransky et al, 1992; Habuchi et al, 1993; Chern et al, 1996) implies that either residual cells have been left at TURBT resulting in tumour recurrence at the original site or that intraluminal shedding and subsequent seeding occurs at the time of TURBT. There are several reports supporting this shedding and seeding hypothesis (Soloway and Masters, 1980; See et al, 1989; Rebel et al, 1994; Fadl-Elmula et al, 1999) and the effect of reduction in recurrence rate following intravesical instillation of various agents at the time of TURBT supports this concept (Lamm and Griffith, 1992). There is a clear need for models in which to investigate the process of bladder cancer seeding and invasion and also upon which to test potential therapeutic agents.

The most commonly used models of invasion are the Boyden chamber and some form of matrigel assay. It is now clear that the extracellular matrix is more than a passive support but interacts with tumour cells regulating differentiation (Fujiyama et al, 1995; Scriven et al, 1997), proliferation (Southgate et al, 1994; Booth et al, 1997) and tumour angiogenesis (Vladovski et al, 1997). Likewise xenograft models are often technically difficult, with moderate levels of reproducibility and are more difficult to control. Therefore although these models have provided useful information (Cook and Hampton, 1997; Miyake et al, 1997) they are becoming inadequate.

We have developed an in vitro model using de-epithelialized rat bladder onto which human bladder cancer cells are seeded, an analogous situation to seeding of cells at TURBT, and used this model to test potential mechanisms and therapeutic inhibitors of invasion.

The plasminogen activator system regulates extracellular matrix degradation and is therefore critical in tumour cell implantation, invasion and metastasis (Conese and Blasi, 1995; Hudson and McReynolds, 1997). The most common plasminogen activator, urokinase plasminogen activator (uPA) functions via its receptor, urokinase plasminogen activator receptor (uPAR). High expression of both uPA and uPAR has been found to be of prognostic significance in a number of tumours including bladder (Hasui et al, 1994; Pedersen et al, 1994; Dickinson et al, 1995). Using this model we have investigated the effects of uPAR antagonists, suramin and N-acetylcysteine. The latter two are compounds that

Received 21 July 2000
Revised 16 November 2000
Accepted 17 November 2000

AJ is supported by the Osman Hill research scholarship from the Royal College of Surgeons of England and the British Urological Foundation. RB and ALH are supported by the Imperial Cancer Research Fund.
*These authors contributed equally to this paper.
The abbreviations used are; VEGF – vascular endothelial growth factor, NAO – N-acetylcysteine, uPA – urokinase plasminogen activator, uPAR – urokinase plasminogen activator receptor, PAI-1 – plasminogen activator inhibitor type 1, TURBT – transurethral resection of bladder tumour.

modify tumour cell–extracellular matrix interactions and therefore have clinical potential.

Suramin is a potent antagonist of the binding of angiogenic factors to their receptors (Pesenti et al, 1992) and an antagonist of uPAR (Behrendt et al, 1993). It has shown anti-tumour activity in prostate cancer, when given systemically, but a narrow therapeutic window resulted in dose-limiting toxic side effects (Eisenberger et al, 1993). There is potential for suramin used intravesically because there should be only limited systemic absorption due to its large molecular weight. Therefore high intravesical concentrations may be achievable (Walther et al, 1996). Not only have anti-angiogenic strategies resulted in reduced tumour growth and vascularity in xenografts (Kim et al, 1993; O'Reilly et al, 1997; Borgstrom et al, 1998) but interference with angiogenesis has also reduced tumour cell invasion (Brooks et al, 1995; Skobe et al, 1997).

The thiol N-acetylcysteine (NAC), interferes with tumour-cell invasion and metastasis, probably due to inhibition of matrix metalloproteinases (Albini et al, 1995). Furthermore NAC is an inhibitor of the Ras oncogene (Irani et al, 1997), demonstrated by transfection experiments, to be important in bladder cancer invasion (Theodorescu et al, 1990). The EJ cell line has a mutated Ha-Ras oncogene (Parada et al, 1982) and therefore we also tested the ability of NAC to inhibit invasion in our model.

Here we report the development and use of a rat bladder in vitro model of bladder cancer invasion, which allows for extracellular matrix interactions. Using this model the therapeutic potential of a range of compounds is illustrated. Based on these findings, and other observations (Pesenti et al, 1992; Behrendt et al, 1993; Eisenberger et al, 1993; Walther et al, 1996), we have started a phase I trial of intravesical suramin in patients with bladder cancer.

### MATERIALS AND METHODS

### Primary culture

The established human transitional cell carcinoma (TCC) cell lines used in this study were RT4, RT112, 253J and EJ28 (gift from Dr M Knowles, Imperial Cancer Research Fund, Leeds, UK). These TCC cell lines display phenotypes associated with well-differentiated, moderately differentiated, and anaplastic, poorly differentiated TCC cells, respectively. Cell lines of truly superficial bladder cancer do not exist. RT4 and RT112, although originally from invasive bladder cancers, behave in a superficial pattern and are widely used as models of superficial bladder cancer (Theodorescu et al, 1990; Redwood et al, 1992; Booth et al, 1997; Davies et al, 1999), 253J and EJ28 are widely used as models of invasive bladder cancer (Elliot et al, 1974; Redwood et al, 1992; Theodorescu et al, 1998; Davies et al, 1999).

Cell lines were cultured in a RPMI (Clare Hall laboratories, Imperial Cancer Research Fund) supplemented with 10% fetal bovine serum (FBS), penicillin G (45 $\mu$g ml$^{-1}$), streptomycin (45 $\mu$g ml$^{-1}$), and kanamycin (90 $\mu$g ml$^{-1}$), at 37°C in a 5% $CO_2$ humidified atmosphere.

### Doubling time of human bladder cancer cell lines

To examine doubling time of these human bladder cancer cell lines, $5 \times 10^4$ cells of each cell line were seeded in 6 well plates, and the number of cells in each cell line were counted in triplicate daily over the next 7 days. Doubling time of each cell line was calculated during the exponential growth phase.

### In vitro invasion assay

6-month-old Sprague-Dawley rats that had served as negative controls in other experiments were used for these experiments. The urinary bladder was removed through a laparotomy incision and transferred to a universal container with sterile culture medium for washing. Bladders were then cut into halves and digested with a 1000 units ml$^{-1}$ bacterial neutral protease (Dispase I, Godo-Shusei Co Ltd, Tokyo, Japan) for 2 hours at room temperature. The urothelium was then stripped off with dissecting forceps and the hemibladders were then placed (de-epithelialized surface face up) onto filter paper. The remaining stroma, with intact basement membrane was then divided further to yield typically 3 sections (approximately 0.5 × 0.5 cm) from each hemibladder (Figure 1). Typically experiments were performed using 6–8 rat bladders. All explants were then pooled so that when each experimental condition was performed in triplicate, each explant was likely to originate from different bladders. Whilst this would increase interindividual variation it was felt that such randomization would minimize spurious effects from any one bladder.

Three 30 mm diameter culture inserts (with a 0.4 $\mu$m pore size nitrocellulose base – Millicell CM, Millipore, Bedford, MA) were placed into a 10 cm culture dish. Onto each culture insert, 0.5 ml of a collagen gel solution was pipetted (1 volume of 10 × concentration RPMI [without $NaHCO_3$], 1 volume of reconstruction buffer [2.2 g $NaHCO_3$ and 4.77 g HEPES in 100 ml 0.05 M NaOH] and 8 volumes of type I collagen solution [Cellmatrix, Nitta Gelatin Co Ltd, Osaka, Japan]). The bladder explants were then removed from the filter paper and placed, de-epithelialized surface uppermost, on the gel (up to 6 explant per insert). This was then placed in the incubator at 37°C for 15–30 minutes to fix the de-epithelialized stroma on the gel.

After 15–30 minutes, cultured cells to be applied were harvested in the standard way with the exception that trypsin was neutralized by mixing with 100% FBS and centrifuging (a process repeated twice) to ensure that no traces of trypsin remained. Cells were then resuspended in medium and the required number ($1 \times 10^5$ RT112, 253J, EJ28 and $1 \times 10^6$ RT4, depending on cell line) was pipetted in 2 ml of complete medium into each 30 mm culture insert. 6 ml of complete medium were then placed in the 10 cm culture dish outside the three 30 mm culture inserts. This protocol (6 ml medium outside three 30 mm culture inserts) was the exact amount of medium to create an air–liquid interface within each culture insert at the level of the explant, which appears essential for correct functioning of the assay. At 24 hours the 2 ml of medium in the 30 mm culture insert was aspirated and not replaced. The medium in the 10 cm culture dish was renewed every 3–4 days, periods longer than this were associated with dehydration of the explants.

### Assessment of invasion

Organ cultures were harvested at 3, 7, 14 and 28 days. Each explant was individually cut out of the insert, fixed in 10% neutralized formalin and embedded in paraffin wax, with triplicates embedded within the same block. Results represent one set of histopathological sections taken from each explant, sectioned near its centre, not multiple sections from different sites within the same explant. Thus repeats are true repeats, representing results from different explants, not just different histopathological sections within the same explant. Histopathological sections

*© 2001 Cancer Research Campaign*

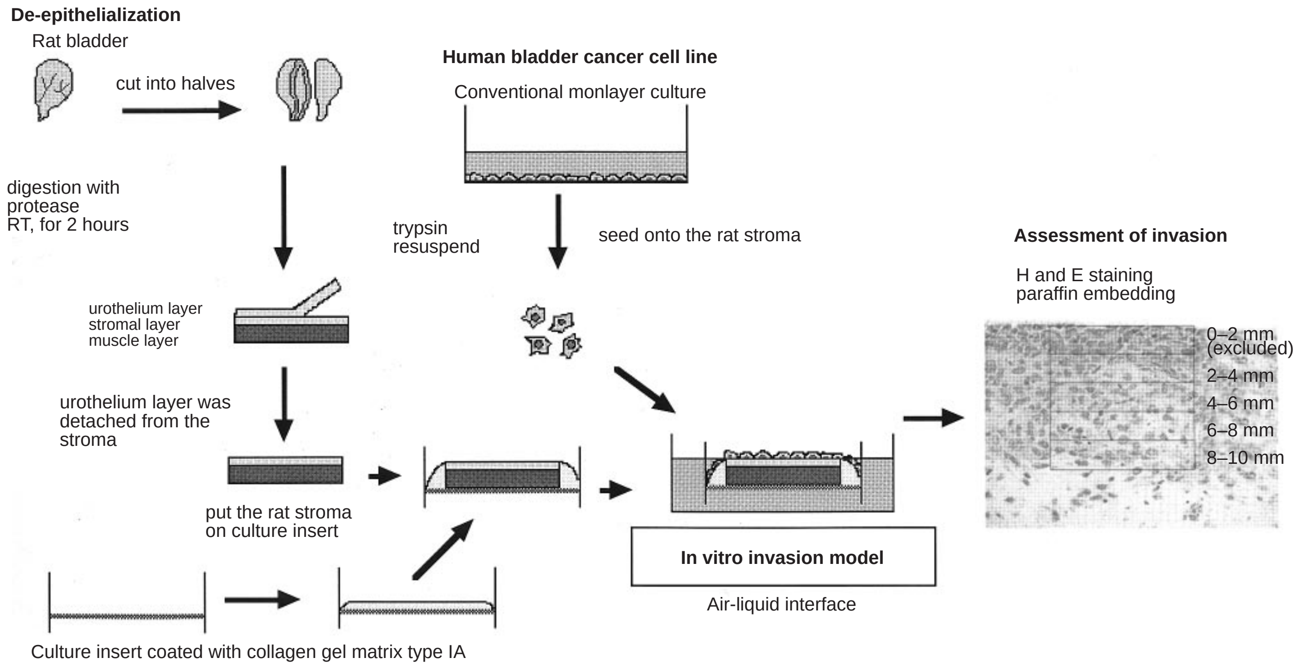

**Figure 1** Experimental procedures of in vitro invasion assay. The left side of the figure demonstrates de-epithelialization of rat bladder. The centre of the figure shows setting up the rat stroma on culture inserts. Human bladder cancer cells are seeded onto this de-epithelialized rat stroma. The right side demonstrates the assessment of invasion. All cells are counted in 2 mm deep rows, starting from a depth of 2 mm, up to 10 mm (RT = room temperature, H and E = haematoxylin and eosin). The grid for quantifying depth of invasion is shown schematically at the bottom right of the figure. In this example there would be 26 cells at depth 2–4 mm and 20 cells at depth 8–10 mm in this field

(5 μm) were dewaxed and rehydrated through graded alcohols to water. For histological evaluation, sections were stained and examined as H and E sections. To quantify invasion, sections were viewed at high power (× 400) under a microscope. A 100, 1 mm × 1 mm, square graticule (Graticules Ltd, Tonbridge, UK) was placed with the top edge along the upper surface of the explant at the left hand edge of the section. To exclude false invasion results due to an undulating upper surface of the rat explant and to account for variable thicknesses of the stratified epithelium, which did not represent invasion, the first 2 rows were not counted. Thereafter all cells were counted in 2 mm deep rows, starting from a depth of 2 mm to 10 mm. The whole section was counted by moving the graticule from left to right. Results for each depth on each section represent the average of at least 10 graticule counts. Results illustrated graphically for each cell line/condition are the means of 4–6 representative rat explants at each depth. Experiments were repeated at least 3 times.

### Immunohistochemical staining

The immunoreactivity of antigens masked by the tissue processing was restored by 10 minutes of digestion in 0.1% trypsin and microwave boiling. Immunolabelling was performed using an indirect streptavidin ‘ABC’ immunoperoxidase method (Dako, High Wycombe, Bucks, UK). Non-specific background staining was blocked by incubation in 10% swine serum. Sections were incubated in primary antibody, against cytokeratin 18 (LP34, Dako), Ki-67 (MIB-1, Beckman Coulter, High Wycombe, Bucks, UK), and laminin (Sigma-Aldrich, Poole, Dorset, UK) and then incubated with a biotin-labelled goat anti-mouse secondary antibody followed by horseradish peroxidase-conjugated streptavidin ABC complex. The peroxidase reaction was developed using diaminobenzidine and slides were washed and mounted in aqueous mountant (Gurr, BDH Lab, Poole, Dorset, UK).

### Addition of inhibitors

N-acetylcysteine (Sigma-Aldrich, Poole, Dorset, England), Å5 a urokinase receptor antagonist (Ångstrom, San Diego, CA, gift of Dr T Jones) and Mab 3936 an inhibitory antibody against urokinase (American Diagnostica, Greenwich, CT) (Hudson and McReynolds, 1997) were assessed for their ability to inhibit invasion. To examine the direct effect of these reagents on the growth of EJ28 cells in monolayer culture, $3 \times 10^3$ cells were seeded onto a 96-well culture plate in 100 μl of conditioned medium with 10% FBS. At 24 hours (day 1) the medium was changed to conditioned medium containing inhibitor at multiple increasing concentrations (from 0–1 mM for N-acetylcysteine and suramin, from 0–5 μM for Å5 and from 0–20 μg $ml^{-1}$ for Mab 3936). On day 4, cell viability was measured using an MTT assay kit (Chemicon International, Temecula, CA) according to the manufacturer’s instructions and confirmed by examination under microscope.

Following the results of these MTT assays, the concentrations of these inhibitors to be tested in the in vitro assay were chosen as 1 mM for NAC and suramin and 5 μM for Å5 and 20 μg $ml^{-1}$ for Mab 3936. These concentrations were chosen because they were non-toxic to our cells in the MTT assay and were able to be well tolerated clinically when given either intravesically (suramin) (Walther et al, 1996) or systemically at much higher concentration (140 mg $kg^{-1}$ body weight – approximately 0.9 mM $kg^{-1}$) for the treatment of paracetomol (acetaminophen) overdose (N-acetylcysteine) (Yip et al, 1998). These inhibitors were added in

 *© 2001 Cancer Research Campaign*

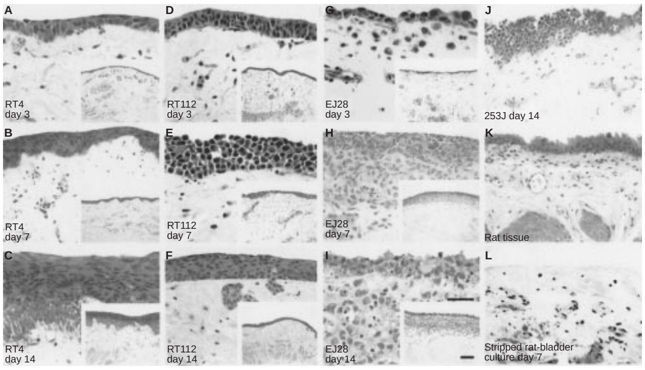

**Figure 2** Time course for growth on in vitro model for superficial tumour cell lines RT4 and RT112 (**A–F**) and invasive tumour cell lines 253J and EJ28 (**G–J**). Both RT4 and RT112 formed stratified epithelium without invasion on the de-epithelialized rat bladder mucosa. RT112 also very occasionally formed a 'nest' of cells, seen in the stroma (**F**). 253J did not invade, but formed a stratified epithelium (**J**), whereas EJ28 invaded diffusely into rat stroma, starting as early as 3 days (**G–I**). **K** demonstrates unstripped rat bladder tissue prior to any experimental procedure. **L** shows de-epithelialized cultured rat bladder used as control. Results show representative histological sections from experiment repeated ≥5 times with ≥3 bladder explants per timepoint. Scale bar: 20 μm. Original magnification × 400. Inset, scale bar: 100 μm. Original magnification × 100

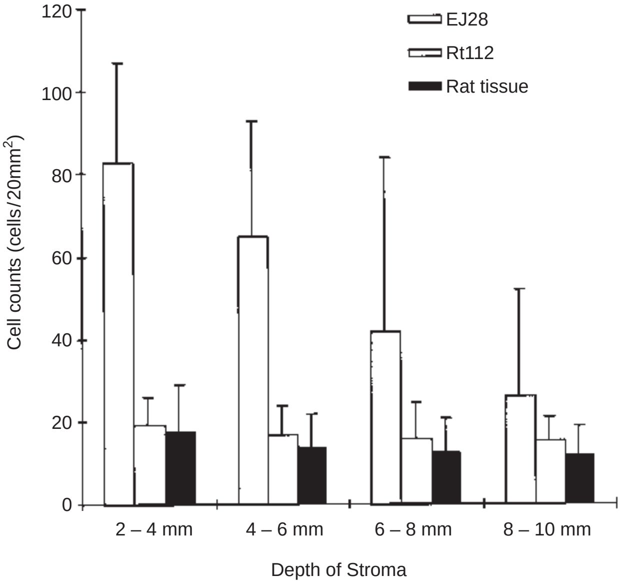

**Figure 3** Quantification of invasion. The number of cells counted in the stroma of control stripped rat tissue and RT112 represent background levels for normal cells, such as fibroblasts and smooth muscle cells. The results for EJ28 includes these background cells, but the higher counts are due to invading bladder cancer cells. Results illustrated from representative experiment (day 14) are means ± S.D. of 10 graticule counts per histological section, 6 explants per cell line. Experiment repeated 3 times. (As RT4 and RT112 were both non-invasive, only 1 cell line was counted, but qualitatively they were the same)

0.1 ml of conditioned medium onto the surface of rat explants from culture day 0 and thereafter every 24 hours. Control bladder explants had medium only added. Separate petri dishes were used for each control or inhibitor group to avoid cross-contamination in the medium.

### Statistical analysis

Statistical analysis was performed using Excel (Microsoft, CA). Comparisons between groups were made using Student's unpaired *t*-test.

### RESULTS

### Growth characteristics of cell lines in vitro on monolayer culture

The 2 superficial cell lines, RT4 and RT112 both had similar but slower mean (± S.D) doubling times than the 2 invasive cell lines, 253J and EJ28, which were similar to each other (RT4 56 h ± 3, RT112 59 h ± 2 Vs 253J 31 h ± 1, EJ28 32 h ± 1, $P = < 0.01$, $n = 3$, experiment repeated 3 times).

### Growth characteristics in the in vitro invasion model

Organ cultures of rat de-epithelialized stroma have produced reproducible results and could be kept viable for more than 28 days, although typically there was little change between 14 and 28 days. The 2 superficial cell lines reproducibly grew in a superficial fashion, forming a stratified epithelium. This pattern was progressive with time (Figure 2A–C RT4, D-F RT112) and no invasion was seen although occasionally (about once in every 10 sections) a 'nest' of cells was seen within vessels in the stroma in RT112 cultures (Figure 2F). Whilst interesting, this is probably not frequent enough to provide a model for vascular invasion.

The 2 invasive lines showed disparate behaviour. 253J formed superficial cell layers and did not invade (Figure 2J) whereas EJ28 invaded diffusely across a broad front. This invasion started as early as 3 days (Figure 2G) and increased with time (Figure 2G–I). In control de-epithelialized bladder explants, i.e. those not reseeded with human bladder cancer cells, there was negligible regrowth of normal rat urothelium (Figure 2I).

### Quantification of invasion

Figure 3 quantifies the depth of invasion according to our method. The number of cells counted in the stroma in control de-epithelialized bladder explant sections and RT112 sections represents background levels for staining of normal stromal cells, such as fibroblasts and smooth muscle cells. These are evenly distributed throughout the stroma. The results for EJ28 include these background cells, but the higher counts are due to invading bladder cancer cells, hence the decrease in number the further away from the upper surface of the bladder. This was confirmed by staining with the specific epithelial marker cytokeratin 18 (data not shown).

### Proliferation of cancer cell lines in vitro model

As demonstrated, the doubling times for 253J and EJ28 were similar in conventional monolayer culture. However, invasion in our in vitro model is unlikely to be simply a manifestation of a faster proliferation rate, since 253J did not invade into the stroma whereas EJ28 did. Furthermore, in the invasion model RT112 and EJ28 had similar proliferation rates as evidenced by Ki-67 immunostaining (data not shown).

© 2001 Cancer Research Campaign 

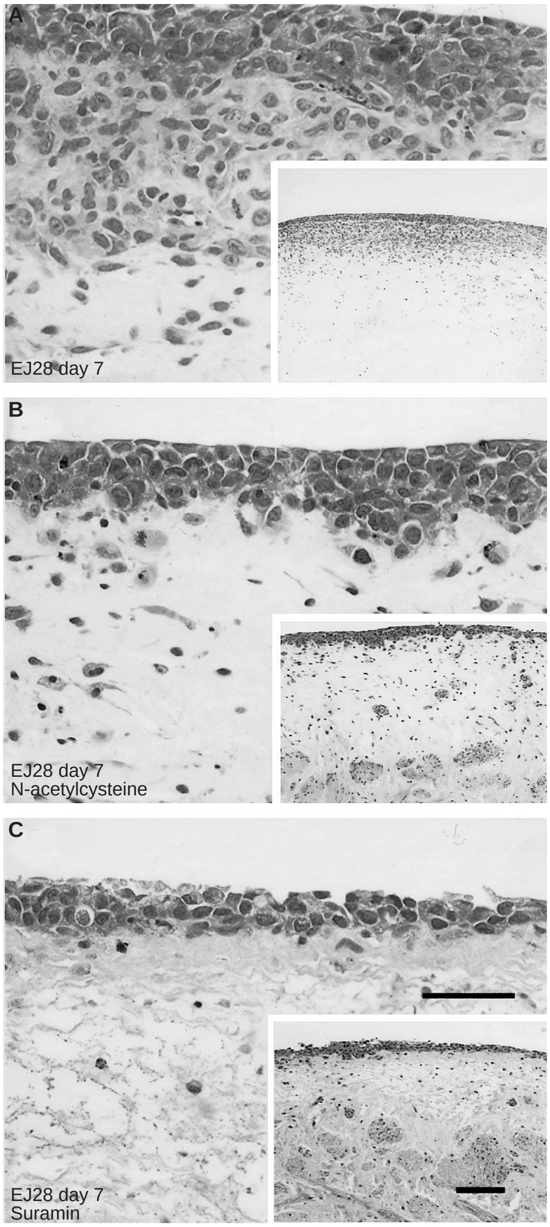

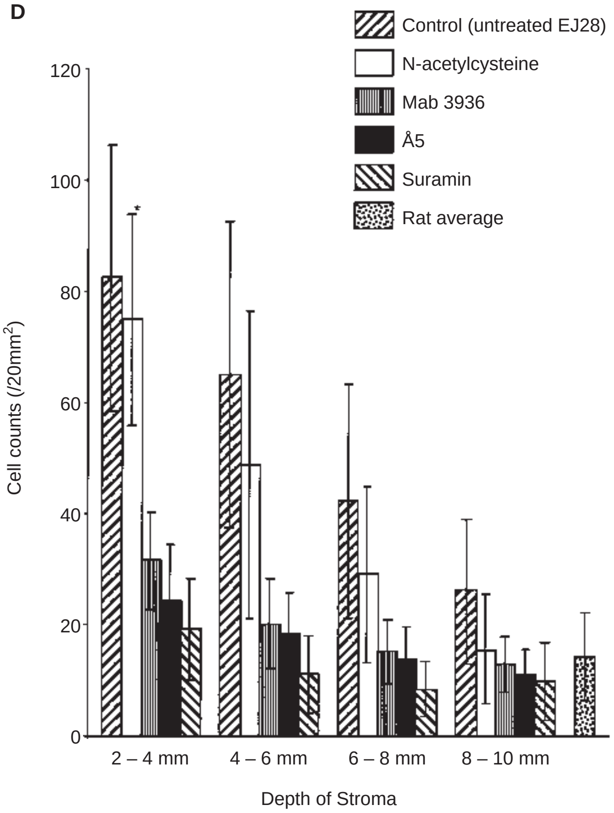

**Figure 4** Inhibition of invasion of EJ28 cells in the in vitro model. (**A**), control untreated EJ28 cells show characteristic invasion. N-acetylcysteine (**B**) and suramin (**C**) inhibited the invasion of EJ28 cells into the rat stroma within 7 days. (**D**) Quantification of invasion. N-acetylcysteine produced the least, although still significant, reduction in invasion. Suramin caused the greatest inhibition of invasion of EJ28 into the rat stroma with uPAR antibody and Å5 intermediate. Results illustrated from representative experiment are means ± S.D. of 10 graticule counts per histological section, 4–6 explants per inhibitor. Experiment repeated 3 times. Scale bar: 20 μm. Original magnification × 400. Inset, scale bar: 100 μm. Original magnification × 100. (*$P$ = 0.057, all other depths for all other inhibitors $P$ = <0.05 vs EJ at that level).

The basement membrane for control rat bladder tissue and RT112 sections showed positive staining with laminin but there was no positive staining in EJ28 sections indicating degradation of the basement membrane.

### Inhibition of invasion

The MTT assay ($n$ = 8 per concentration, experiment repeated twice) showed no growth inhibition of N-acetylcysteine and suramin on EJ28 cells up to 1 mM for 4 days and no inhibition by Å5 up to 5 μM or by Mab 3936 up to 20 μg ml$^{-1}$. However in the in vitro invasion assay the invasion of EJ28 cells into the rat stroma was significantly inhibited by suramin and N-acetylcysteine ($P$ < 0.05). Suramin had the greatest effect with almost complete inhibition of invasion by EJ28 cells (Figure 4), whilst, the least inhibition was seen with N-acetylcysteine (Figure 4).

### Inhibition of components of the plasminogen activator system

Inhibition of the plasminogen activator system blocked EJ28 invasion effectively with Å5 and Mab 3936 producing inhibition equivalent to each other and to suramin ($P$ = 0.05 to 0.01 versus control, but not significantly different from each other, unpaired $t$ test).

### DISCUSSION

Our model, using de-epithelialized rat bladder to expose the underlying stroma allows for these tumour cell–extracellular matrix interactions and more direct analysis of new drugs or biochemical mechanisms. We studied 4 cell lines that demonstrate phenotypes associated with well, moderate and poorly differentiated TCC

© 2001 Cancer Research Campaign cells. RT4 and RT112 have been used as models of superficial non-invasive tumours and 253J and EJ28 are models of invasive bladder cancer (Elliot et al, 1974; Theodorescu et al, 1990; Redwood et al, 1992; Booth et al, 1997; Theodorescu et al, 1998; Davies et al, 1999). Although reported as invasive, 253J was not invasive in our model. This probably confirms the importance of multiple interactions for invasion to take place, one or more of which were missing for 253J to invade. In our model since EJ28 invaded, the cell line was used to investigate the effects of various inhibitors.

Urokinase plasminogen activator (uPA) and urokinase plasminogen activator receptor (uPAR) correlated with invasion into matrigel by bladder tumour cell lines in a previous study (Hudson and McReynolds, 1997). Our study differed in that we studied a complex stroma with viable cells, connective tissue, blood vessels and analysed other mechanisms for modifying uPA effects. The inhibition in our model with Mab 3936 confirmed the importance of the plasminogen activator system in a realistic model of bladder cancer invasion and pinpoints a potential therapeutic target (Ossowki et al, 1991). We also tested Å5, a small cyclic peptide inhibitor of uPAR, and found it was also able to block invasion. This shows that uPA must associate with its cell surface receptor for bladder tissue invasion and secretion is insufficient. Such strategies may be useful after TURBT to prevent displaced cells seeding.

A concentration of 1 mM suramin was able to completely inhibit invasion. This level cannot be achieved systemically, but in a phase 1 trial, it was found possible to achieve this level safely by intravesical instillation. Thus further evaluation is justified.

Booth et al have previously reported an in vitro model using human urinary bladder (Booth et al, 1997). The latter is an excellent model and human tissue obviously has advantages over rat tissue when investigating human bladder cancer invasion, although our model also has specific advantages. Firstly, it is harder to maintain a regular supply of human bladder for experiments. Secondly, the viability of these models depends on diffusion of culture medium and the maintenance of an air–liquid interface. This is relatively straightforward in the thinner rat bladder, which can be kept viable for more than 28 days. Human bladder cancer cell lines remain viable on rat de-epithelialized stroma, because there is no immunological rejection in vitro. Marked invasion into the rat stroma occurs within a short time, 7–14 days. This shorter experimental time, ease of handling and plentiful supply means that this model will aid the investigation of the mechanism of invasion and the testing of therapeutic agents. This would become important if larger studies were to be done, for example, investigating reversibility of effect with intermittent treatment similar to weekly intravesical instillations.

In conclusion, this study illustrates a reproducible and realistic model of bladder cancer invasion. It has enabled a target concentration of suramin for a phase I trial to be determined. It allows for the study of the mechanisms involved in invasion. These processes and the contribution of the stroma could be further elucidated by using bladders from transgenic animals deficient in specific components of the extracellular matrix.

### ACKNOWLEDGEMENTS

We thank Prof J Southgate and her team for helpful advice on establishing the model, Dr Simon Biddolph and the staff of the Churchill Research Institute for their technical help.

### REFERENCES

Albini A, D'Agostini F, Giunciuglio D, Paglieri I, Balansky R and De Flora S (1995) Inhibition of invasion, gelatinase activity, tumour take and metastasis of malignant cells by N-acetylcysteine. *Int J Cancer* **61**: 121–129

Bajour K, Noel A, Gerard RD, Masson V, Brunner N, Holst-Hansen C, Skobe M, Fusenig NE, Carmeliet P, Collen D and Foidart JM (1998) Absence of host plasminogen activator inhibitor 1 prevents cancer invasion and vascularisation. *Nature Med* **4**: 923–928

Behrendt N, Ronne E and Dano K (1993) Binding of the urokinase-type plasminogen activator to its cell surface receptor is inhibited by low doses of suramin. *J Biol Chem* **268**: 5985–5989

Bhat GJ, Gunaje JJ and Idell S (1999) Urokinase-type plasminogen activator induces tyrosine phosphorylation of a 78-kDa protein in H-157 cells. *Am J Physiol* **277**: L301–L309

Booth C, Harnden P, Trejdosiewicz LK, Scriven S, Selby PJ and Southgate J (1997) Stromal and vascular invasion in an human in vitro bladder cancer model. *Lab Invest* **76**: 843–857

Borgstrom P, Bourdon MA, Hillan KJ, Sriramarao P and Ferrara N (1998) Neutralising anti vascular endothelial growth factor antibody completely inhibits angiogenesis and growth of human prostate carcinoma microtumours in vivo. *Prostate* **35**: 1–10

Brooks PC, Stromblad S, Klemke R, Visscher D, Sarkar FH and Cheresh DA (1995) Antiintegrin $\alpha v\beta 3$ blocks human breast cancer growth. *J Clin Invest* **96**: 1815–1822

Chern H-D, Becich MJ, Persad RA, Romkes M, Smith P, Collins C, Li Y-H and Branch RA (1996) Clonal analysis of human recurrent superficial bladder cancer by immunohistochemistry of P53 and retinoblastoma proteins. *J Urol* **156**: 1846–1849

Conese M and Blasi F (1995) The urokinase/urokinase-receptor system and cancer invasion. *Baillières Clin Haematol* **8**: 365–389

Cook GP and Hampton JA (1997) Effects of ibuprofen on the in vitro invasiveness of a human transitional cell carcinoma. *Anticancer Res* **17**: 365–368

Crew JP, O'Brien T, Bradburn M, Fuggle S, Cranston D and Harris AL (1997) Vascular endothelial growth factor is a predictor of relapse and stage progression in superficial bladder cancer. *Cancer Res* **57**: 5281–5285

Davies G, Jiang WG and Mason MD (1999) Cell–cell adhesion molecules and their associated proteins in bladder cancer cells and their role in mitogen induced cell–cell dissociation and invasion. *Anticancer Res* **19**: 547–552

Dickinson AJ, Savage PB, Newcomb PV, Lodge R and Sibley GN (1995) The expression of urokinase, its receptor and plasminogen activator inhibitor-1 in bladder cancer (abstract). *J Urol* **153**: 406A

Eisenberger M, Reyno LM, Jodrell DI, Sinibaldi VJ, Tkaczuk KH, Sridhara R, Zuhowski EG, Lowitt MH, Jacobs SC and Egorin MJ (1993) Suramin, an active drug for prostate cancer: interim observations in a phase I trial. *J Natl Cancer Inst* **85**: 611–621

Elliot AY, Cleveland P, Cervenka J, Castro AE, Stein N, Hakala TR and Fraley EE (1974) Characterisation of a cell line from human transitional cell cancer of the urinary tract. *J Natl Cancer Inst* **53**: 1341–1349

Fadl-Elmula I, Gorunova L, Mandahl N, Elfving P, Lundgren R, Mitelman F and Heim S (1999) Cytogenetic monoclonality in multifocal uroepithelial carcinomas: evidence of intraluminal tumour seeding. *Br J Cancer* **81**: 6–12

Folkman J (1990) What is the evidence that tumours are angiogenesis dependent? *J Natl Cancer Inst* **82**: 4–6

Fujiyama C, Masaki Z and Sugihara H (1995) Reconstruction of the urinary bladder mucosa in three dimensional collagen gel culture: Fibroblast-extracellular matrix interaction of transitional epithelial cells. *J Urol* **153**: 2060–2067

Gilbert HA, Logan SL, Kagan AR, Friedman HA, Cove JK, Fox M, Muldoon TM, Lonni YW, Rowe JH, Cooper JF, Nussbaum H, Chan P, Rao A and Starr A (1978) The natural history of papillary transitional cell carcinoma of the bladder and its treatment in an unselected population on the basis of histologic grading. *J Urol* **119**: 488–492

Grondhal-Hansen J, Christensen IJ, Briand P, Pappot H, Mouridsen HT, Blichert-Toft M, Dano K and Brunner N (1997) Plasminogen activator inhibitor type 1 in cytosolic tumour extracts predicts prognosis in low-risk breast cancer patients. *Clin Cancer Res* **3**: 233–239

Habuchi T, Takahashi R, Yamada H, Kakehi Y, Sugiyama T and Yoshida O (1993) Matachronous multifocal development of urothelial cancers by intraluminial seeding. *Lancet* **342**: 1087–1088

Harris AL and Neal DE (1992) Bladder cancer-field versus clonal origin. *N Engl J Med* **326**: 759–761

Hasui Y, Marutsuka K, Nishi S, Kitada S, Osada Y and Sumiyoshi A (1994) The content of urokinase-type plasminogen activator and tumour recurrence in superficial bladder cancer. *J Urol* **151**: 16–20

© 2001 Cancer Research Campaign

Hudson ML and McReynolds LM (1997) Urokinase and the urokinase receptor: Association with in vitro invasiveness of human bladder cancer cell lines. *J Natl Cancer Inst* **89**: 709–717

Irani K, Xia Y, Zweier JL, Sollott SJ, Der CJ, Fearon ER, Sundaresan M, Finkel T and Goldschmidt-Clermont PJ (1997) Mitogenic signaling mediated by oxidants in Ras-transformed fibroblasts. *Science* **275**: 1649–1652

Kim KJ, Li B, Winer J, Armanini M, Gillett N, Phillips HS and Ferrara N (1993) Inhibition of vascular endothelial growth factor-induced angiogenesis suppresses tumour growth in vivo. *Nature* **362**: 841–844

Lamm DL and Griffith JG (1992) Intravesical therapy: Does it affect the natural history of superficial bladder cancer? *Semin Urol* **10**: 39–44

Liotta LA, Rao CN and Barsku SH (1983) Tumour invasion and the extracellular matrix. *Lab Invest* **49**: 636–649

Ma D, Gerard RD, Li XY, Alizadeh H and Niederkorn JY (1997) Inhibition of metastasis of intraocular melanomas by adenovirus-mediated gene transfer of plasminogen activator inhibitor type 1 (PAI-1) in an athymic mouse model. *Blood* **90**: 2738–2746

Miyake H, Yoshimura K, Hara I, Eto H, Arakawa S and Kamidono S (1997) Basic fibroblast growth factor regulates matrix metalloproteinases production and in vitro invasiveness in human bladder cancer cell lines. *J Urol* **157**: 2351–2355

O'Reilly MS, Boehm T, Shing Y, Fukai N, Vasios G, Lane WS, Flynn E, Birkhead JR, Olsen BR and Folkman J (1997) Endostatin: An endogenous inhibitor of angiogenesis and tumour growth. *Cell* **88**: 277–285

Oshinsky GS, Chen Y, Jarett T, Anderson AE and Weiss GH (1995) A model of bladder tumour xenografts in the nude rat. *J Urol* **154**: 1925–1929

Ossowski L (1992) Invasion of connective tissue by human carcinoma cell lines: requirement for urokinase receptor, and interstitial collagenase. *Cancer Res* **52**: 6754–6760

Ossowki L, Russo-Payne H and Wilson EL (1991) Inhibition of urokinase-type plasminogen activator by antibodies: the effect on dissemination of a human tumour in the nude mouse. *Cancer Res* **51**: 274–281

Parada LF, Tabin CJ, Shih C and Weinber RA (1982) Human EJ bladder carcinoma oncogene is homolgue of Harvey sarcoma virus ras gene. *Nature* **297**: 474–478

Pedersen H, Brunner N, Francis D, Osterlind K, Ronne E, Hansen HH, Dano K and Grondahl-Hansen J (1994) Prognostic impact of urokinase, urokinase receptor, and type 1 plasminogen activator inhibitor in squamous and large cell lung cancer tissue. *Cancer Res* **54**: 4671–4675

Pesenti E, Sola F, Mongelli N, Grandi M and Spreafico F (1992) Suramin prevents neovascularisation and tumour growth through blocking of basic fibroblast growth factor activity. *Br J Cancer* **66**: 367–372

Pyke C, Kristensen P, Ralfkiaer E, Grondahl-Hansen J, Eriksen J, Blasi F and Dano K (1991) Urokinase-type plasminogen activator is expressed in stromal cells and its receptor in cancer cells at invasive foci in human colon adenocarcinomas. *Am J Pathol* **138**: 1059–1067

Raghavan D, Shipley WU, Garnick MB, Russell PJ and Richie JP (1990) Biology and management of bladder cancer. *N Engl J Med* **322**: 1129–1138

Rebel JM, Thijssen C, Vermey M, Delouvee A, Zwarthoff EC and Van der Kwast TH (1994) E-cadherin expression determines the mode of replacement of normal urothelium by human bladder carcinoma cells. *Cancer Res* **54**: 5488–5492

Redwood SM, Liu BC, Weiss RE, Hodge DE and Droller MJ (1992) Abrogation of the invasion of human bladder tumour cells by using proetease inhibitor(s). *Cancer* **69**: 1212–1219

Scriven S, Booth C, Thomas DFM, Trejdosiewicz LK and Southgate J (1997) Reconstitution of human urothelium from monolayer cultures. *J Urol* **158**: 1147–1152

See WA, Miller JS and Williams RD (1989) Pathophysiology of transitional tumour cell adherence to sites of urothelial injury in rats: mechanisms mediating intravesical recurrence due to implantation. *Cancer Res* **49**: 5414–5418

Sidransky D, Frost P, Von Eschenbach A, Oyasu R, Preisinger AC and Vogestein B (1992) Clonal origin of bladder cancer. *N Engl J Med* **326**: 737–740

Silverman DT, Rothman N and Devesa SS (1999) Epidemiology of bladder cancer. In: *Bladder Cancer: Biology, Diagnosis and Management*, Syrigos KN and Skinner DG (eds), pp. 11–55. Oxford University Press: Oxford

Skobe M, Rockwell P, Goldstein N, Vosseler S and Fusenig NE (1997) Halting angiogenesis suppresses carcinoma cell invasion. *Nature Med* **3**: 1222–1227

Soloway MS and Masters S (1980) Urothelial susceptibility to tumour cell implantation – influence of cauterization. *Cancer* **46**: 1158–1163

Southgate J, Hutton KAR, Thomas DFM and Trejdosiewicz LK (1994) Normal human urothelial cells in vitro: proliferation and induction of stratification. *Lab Invest* **71**: 583–594

Theodorescu D, Cornil I, Fernandez BJ and Kerbel RS (1990) Overexpression of normal and mutated forms of HRAS induces orthopic bladder invasion in human transitional cell carcinoma. *Proc Natl Acad Sci USA* **87**: 9047–9051

Theodorescu D, Laderoute KR and Gulding KM (1998) Epidermal growth factor receptor-regulated human bladder cancer motility is in part a phosphatidylinositol 3-kinase-mediated process. *Cell Growth Differ* **9**: 919–928

Vladovski I, Miao H-Q, Benezra M, Lider O, Bar-Shavit R, Schmidt A and Peretz T (1997) Involvement of the extracellular matrix, heparan sulphate proteoglycans, and heparan sulphate degrading enzymes in angiogenesis and metastatis. In: *Tumour angiogenesis*, Bicknell R, Lewis CE and Ferrara N (eds), pp. 125–140. Oxford University Press: Oxford

Wallace DMA, Smith JHF, Billington S, Smith MR, Stemplewski HE and Tipton PW (1984) Promotion of bladder tumours by endoscopic procedures in an animal model. *Br J Urol* **56**: 658–662

Walther MM, Figg WD and Lineham WM (1996) Intravesical suramin: a novel agent for the treatment of superficial transitional-cell carcinoma of the bladder. *World J Urology* **14**: S8–S11

Yip L, Dart RC and Hurlbut KM (1998) Intravenous administration of oral N-acetylcysteine. *Crit Care Med* **26**: 40–43

© 2001 Cancer Research Campaign
